# The effect of botulinum toxin type A in different dilution on the contraction of fibroblast—In vitro study

**DOI:** 10.1111/jocd.13058

**Published:** 2019-07-22

**Authors:** Rungsima Wanitphakdeedecha, Arisa Kaewkes, Chanida Ungaksornpairote, Saowalak Limsaengurai, Uraiwan Panich, Woraphong Manuskiatti

**Affiliations:** ^1^ Department of Dermatology, Faculty of Medicine Siriraj Hospital Mahidol University Bangkok Thailand; ^2^ Department of Pharmacology, Faculty of Medicine Siriraj Hospital Mahidol University Bangkok Thailand

**Keywords:** botulinum toxin A, dilution, fibroblast contraction

## Abstract

**Background:**

Botulinum toxin type A (BoNT‐A) may directly remodel dermal tissues or induce a loss of normal morphology and cytoplasmic retraction and spread. Intradermal injection was claimed to produce a dermo‐lifting effect, including midface lifting by using low concentration with variable dilution.

**Objective:**

To understand how intradermal BoNT‐A achieves tissue lifting, we examined different type of BoNT‐A and their effects on dermal fibroblast contraction.

**Methods:**

Normal human dermal fibroblasts were treated with onabotulinumtoxin (ONA), abobotulinumtoxin (ABO), prabotulinumtoxinA (PRABO), incobotulinumtoxinA (INCO), and letibotulinumtoxin A (LETI) in dilutions used in real‐world practice. Fifty fibroblasts per dilution were photographed and measured the length to demonstrate their contraction every 2 hours from baseline (0 hours) to 12 hours post‐treatment.

**Results:**

ONA did not significantly decrease fibroblast lengths, at any timepoint or dilution. At 1:7 dilution ratios, ABO decreased fibroblast lengths after 2 hours and significantly after 10‐12 hours. At 1:7, 1:8, 1:9, and 1:10 dilution, PRABO decreased length, and most rapidly at 1:7 and 1:8. At 1:6, 1:8, 1:9, and 1:10 dilution, INCO decreased lengths almost immediately. At 1:6 dilution, INCO decreased lengths almost immediately. At 1:7 dilution, INCO decreased lengths after 2‐4 hours, while at 1:8, 1:9, and 1:10 dilution, INCO decreased lenghts nearly imediately. LETI decreased lengths at all dilutions except 1:9, with near‐immediate effects at 1:6, 1:7, 1:8, and 1:10. At 1:4 dilution, LETI decreased lengths from 1 hour.

**Conclusions:**

Different commercial preparations of BoNT‐A toxins cause different fibroblast contractions in vitro. Product selection and dilution used may affect the clinical outcome of intradermal injection of BoNT‐A for face lifting.

## 1 INTRODUCTION

Botulinum toxin type A has been used to treat noncosmetic indications including cervical dystonia, blepharospasm, strabismus, hyperhidrosis, and^1^ eccrine gland abnormalities, such as multiple eccrine hidrocystomas,^2^ Raynaud phenomenon,^3^ and cutaneous leiomyomas.^4^ However, BoNT‐A is most well‐known among the general public for its effects in esthetic medicine, as a result of its ability to relax facial muscles and improve cutaneous elasticity, pliability, and viscoelasticity, and to re‐organize and re‐orientate facial collagen fibers.^5^ For dermal indications, BoNT‐A has been used to change skin texture and sebum production at the site of injection 6, 7 to resolve severe cystic acne,^8^ and reduce sebum production and pore size in patients with oily skin.^9^

Several studies have demonstrated the biological impact of BoNT‐A in directly targeting nonneuronal cell types^10^ including skin cells and tissues that express at least one BoNT‐A‐binding proteins, such as the SV2 vesicular protein or FGFR3.^11^ Some dermal cells may also express the BoNT‐A cleavage target, SNAP‐25, including epidermal keratinocytes and subcutaneous adipose tissue mesenchymal stem cells.^12^ BoNT‐A remodels connective dermal tissue and its cutaneous effects are exploited in cutaneous flaps where it produces specific biological responses in dermal fibroblasts. This includes the expression of cytokines and growth factors such as vascular endothelial growth factor, platelet endothelial cell adhesion molecule 1, CD31, CD34, interleukin (IL)‐1, and tumor necrosis factor‐a.^13^, ^14^, ^15^ In dermal fibroblasts, BoNT‐A may directly facilitate tissue remodeling, wound closure, and scar formation. BoNT‐A induced a loss of normal fibroblast morphology and cytoplasmic retraction and spread in experiments on cultured 3T3 fibroblasts.^16^ It decreased senescence‐related proteins in human dermal fibroblasts exposed to ultraviolet B radiation and induced premature senescence in anti‐photoaging studies. These fibroblasts subsequently had less matrix metalloproteinase (MMP)‐1 and MMP‐3 but more collagen types I (Col‐I) and III (Col‐III). 17 In wound healing experiments on cultured human fibroblasts, BoNT‐A prevented Col‐I and Col‐III expressions but improved MMP‐2 and MMP‐9 expressions, 18 although this observation requires clarification as others have shown the opposite that BoNT‐A upregulated Col‐I expression but decreased MMP expression. 19 BoNT did not, however, stimulate dermal fibroblast proliferation or cause inflammatory effects.

BoNT‐A also inhibited Smad2 phosphorylation during silicone implant capsule formation, while inhibited TGF‐1 signaling to disrupt fibroblast‐to‐myofibroblast differentiation.^20^ Rat injury models demonstrated reduced wound and graft contraction following BoNT‐A treatment, and improved and faster healing, and less severe scarring of burn wounds, with faster regeneration, less inflammation, and more fibroblasts.^21^, ^22^ Animal studies showed reduced hypertrophic scars thickness^23^ due to BoNT‐A modifying fibroblast growth and differentiation. In human fibroblasts, BoNT‐A upregulated Rac1, Cdc42, and RhoA^24^; inhibited fibroblast proliferation and fibroblast‐to‐myofibroblast differentiation^25^; and stimulated apoptosis, but reduced myosin expression. It also regulated Col‐I but downregulated TGF‐b1, VEGF, MMP‐1, and PDGFA and other genes involved in invasive proliferation of keloid fibroblasts.^26^, ^27^, ^28^ At a clinical level, a slight increase in type I procollagen has been observed following the use of abobotulinumtoxin (ABO).^29^

In the cosmetic treatment of hyperfunctional facial lines induced by muscle hyperactivity, BoNT‐A is typically delivered by intramuscular injection.^30^ However, reports of intradermal BoNT‐A inducing a dermo‐lifting effect, such as midface lifting, have surfaced^31^ even with the use of different forms of the toxin (ABO and onabotulinumtoxin (ONA)).^32^ Intradermal BoNT‐A injections in facial rejuvenation can correct the downward pull of midfacial depressor,^29^ platysmal, and lateral orbicularis oculi muscles. Interestingly, toxin interventions in platysmal and lateral orbicularis oculi muscles also increase the lifting effect of the levators to produce the visible midface lift. Our group has also previously demonstrated a significant face‐lifting effect following the use of ABO in a split‐face investigation.^33^ This lifting effect was not physically induced by the pricking of the toxin‐delivery needle^34^ as more recent investigations indicate better clinical improvements with BoNT‐A than with normal saline and an effect at the dermal level. As stated above, one potential mechanism for this lifting may have been the stimulation of collagen production.^5^, ^7^, ^35^ Frontalis lifting has also been achieved by injecting ABO in the hair‐bearing areas of the scalp, in the origin of the frontalis and in the glabella.^36^

However, the mechanism by which intradermal BoNT‐A achieves this lifting effect, aside from its capacity to block muscle‐contracting nerves, remains unclear. Several investigators have proposed mechanisms including the paralysis of depressor muscles, the increase in collagen synthesis, and fibroblast contraction.^2^, ^19^ Whether and how intradermal BoNT‐A injections induce fibroblast contraction, and which toxin types or dilutions potentially achieve this effect, remain to be seen. We therefore set out to examine the effect of BoNT‐A on dermal fibroblast contraction.

## 2 METHODS AND MATERIALS

### 2.1 Cell culture

Normal human dermal fibroblasts (NHDFs; LONZA) derived from adult female skin were cultured in Dulbecco's Modified Eagle Medium (DMEM; Invitrogen) supplemented with 10% fetal bovine serum (Invitrogen), 1% penicillin (100 units/mL; Sigma‐Aldrich), and streptomycin (100 μg/mL; Sigma‐Aldrich) and were maintained at 37°C in a humidified Forma Scientific CO_2_ Water‐Jacketed Incubator (Thermo Scientific Forma), with 5% CO_2_ (PCO2 = 40 Torr). Upon reaching 80%‐100% confluency in a 75 cm^2^ tissue culture flask (Corning), cells were washed with phosphate‐buffered saline, trypsinized (Sigma‐Aldrich), into a single cell suspension. The suspension was washed twice with 10% DMEM to remove trypsin by centrifuging at 1500 rpm for 5 minutes and discarding the supernatant. The cell pellet was resuspended in 10% DMEM, and cells were counted on a haemocytometer. 5 × 10^4^ cells/well were seeded in 6‐well plate and cultured for 24 hours.

### 2.2 Sample preparation and testing

**Figure 1:**
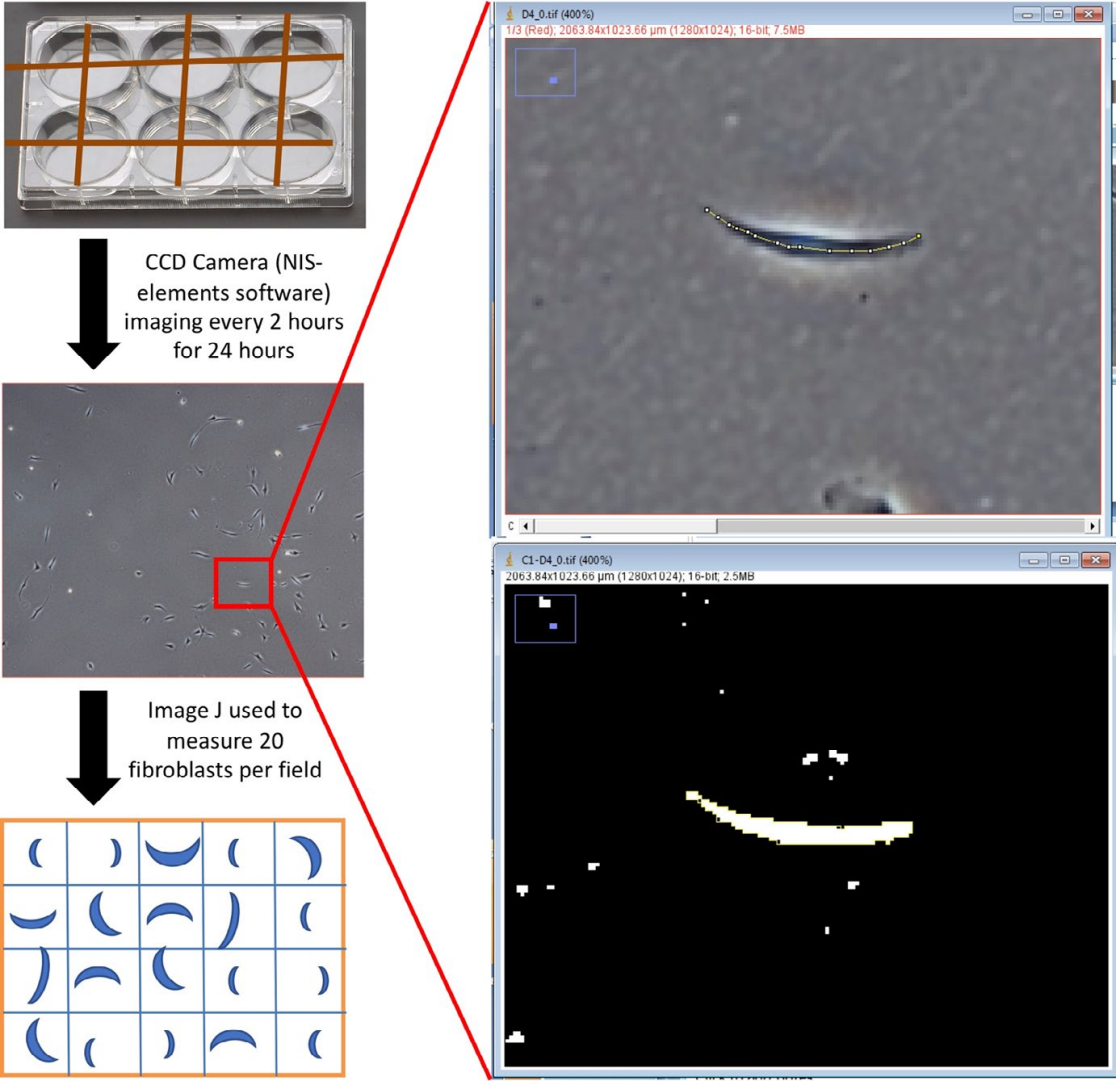
Measurement of fibroblasts. (Left) Fibroblasts were photographed with NIS‐elements imaging software at baseline and every 2 h for 24 h after the intervention. Twenty fibroblasts per field were then measured using the imaging software, image J (right vertical panel). Image J was used to calculate cell length (top) and area (bottom) to determine the contraction of selected dermal fibroblasts

Normal saline (NSS) and 250 μmol/L hydrogen peroxide (H_2_O_2_) solutions were used as a negative and positive control, respectively. The cultured fibroblasts were mixed with five different BoNT‐A: onabotulinumtoxinA (ONA; Botox^®^, Allergan Inc.), abobotulinumtoxinA (ABO; Dysport^®^, Ipsen Biopharm Ltd.), prabotulinumtoxinA (PRABO; Nabota^®^, Daewoong Pharmaceutical), incobotulinumtoxinA (INCO; Xeomin^®^, Merz Pharmaceuticals, GmbH), and letibotulinumtoxinA (LETI; Botulax, Hugel Inc.) in dilutions that are routinely used by the authors in their real‐world clinical practice (Table 1). Photographic documentation of fibroblast length were collected at baseline, immediately after mixing the solution into fibroblast well, 2‐, 4‐, 6‐, 8‐, 10‐, and 12‐hour after mixing. Fifty fibroblasts were randomly selected from each dilution, and photographs of each cell were taken with NIS‐elements imaging software, at baseline (0 hours), immediately after mixing, and every 2 hours until 12 hours after the intervention. Finally, the Image J software 37 was used to measure the lengths of 50 fibroblasts. The mean fibroblast length per timepoint was then used to construct graphs that depict the impact of toxin treatment over the 12‐hour duration. Of note, only 20 fibroblasts could be captured per field of view (see Figure 1).

**Table 1:** Different types and dilutions of botulinum toxin A used in this study

Botulinum toxin A	Dilution	Toxin (unit vial)	NSS (cc)
OnabotulinumtoxinA	1:2.5	100 u	2.5
	1:3	100 u	3.0
	1:3.5	100 u	3.5
	1:4	100 u	4.0
	1:4.5	100 u	4.5
	1:5	100 u	5.0
AbobotulinumtoxinA	1:5	500 u	5.0
	1:6	500 u	6.0
	1:7	500 u	7.0
	1:8	500 u	8.0
	1:9	500 u	9.0
	1:10	500 u	10.0
PrabotulinumtoxinA	1:5	100 u	5.0
	1:6	100 u	6.0
	1:7	100 u	7.0
	1:8	100 u	8.0
	1:9	100 u	9.0
	1:10	100 u	10.0
IncobotulinumtoxinA	1:6	100 u	6.0
	1:7	100 u	7.0
	1:8	100 u	8.0
	1:9	100 u	9.0
	1:10	100 u	10.0
LetibotulinumtoxinA	1:4	100 u	4.0
	1:5	100 u	5.0
	1:6	100 u	6.0
	1:7	100 u	7.0
	1:8	100 u	8.0
	1:9	100 u	9.0
	1:10	100 u	10.0

## 3 RESULTS

**Figure 2:**
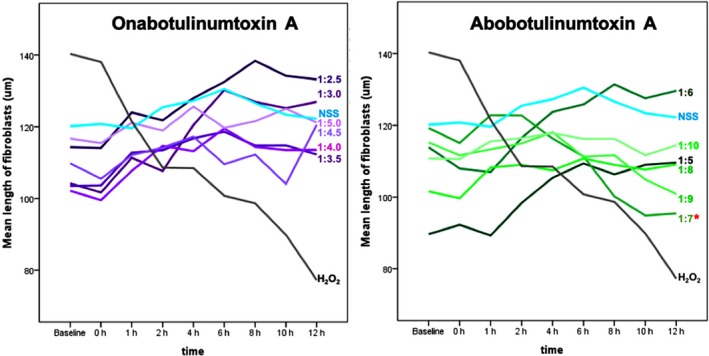
Mean length of fibroblasts over 12 h following ONA treatment (left) or ABO treatment (right). NSS and H_2_O_2_ were used as positive and negative controls

Fibroblasts treated with toxins were measured at each two‐hourly timepoint over a duration of 12 hours. The positive control treatment with H_2_O_2_ significantly decreased fibroblast length (Figure 2) at any dilution, whereas fibroblasts treated with normal saline did not demonstrate any significant contraction at all evaluation timepoints. Compared with the positive control, no dilution of ONA caused a significant decrease in the mean fibroblast length, at any of the tested timepoints.

**Figure 3:**
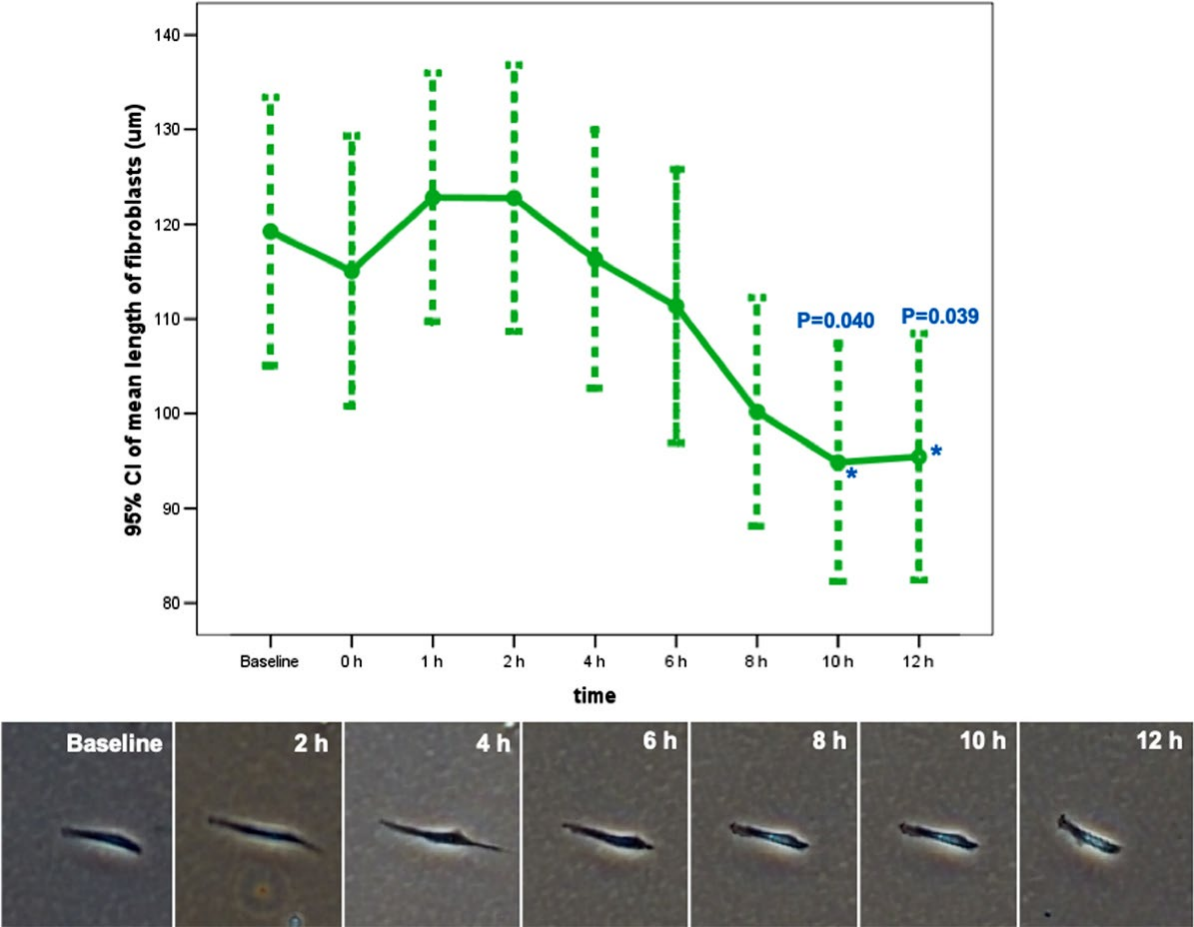
Response of fibroblasts to ABO. Mean length of ABO‐treated fibroblasts over a 12‐hour period (top). Shortening of fibroblasts over a 12 h period (bottom). NSS and H_2_O_2_ were used as positive and negative controls

However, in fibroblasts treated with a 1:7 dilution of ABO, a decrease in fibroblast length commenced 2 hours post‐treatment and became significant between 10‐12 hours post‐treatment (Figures 2 and 3). ABO did not decrease fibroblast lengths at all timepoints when used at 1:5, 1:6, 1:8, 1:9, and 1:10 dilutions.

**Figure 4:**
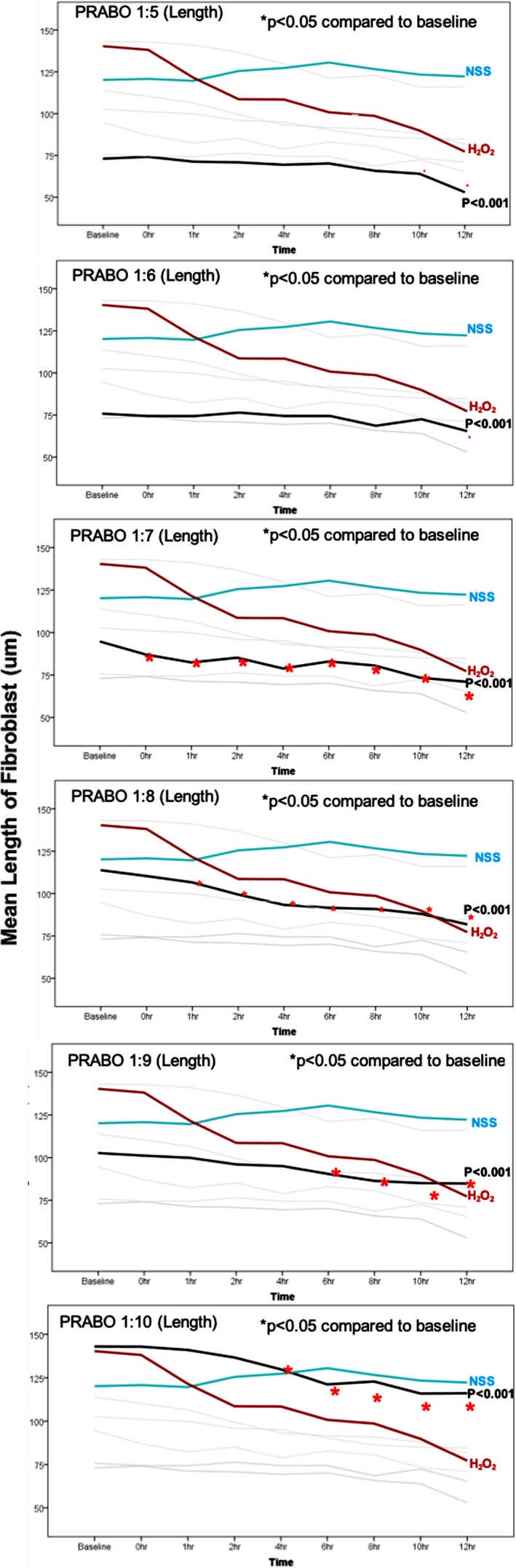
Mean length of PRABO‐treated fibroblasts. PRABO was diluted 1:5, 1:6, 1:7, 1:8, 1:9, and 1:10 and analyzed together with fibroblasts treated with NSS or H_2_O_2_ controls

PRABO (Figure 4) caused a decrease fibroblast length when used at a 1:7, 1:8, 1:9, and 1:10 dilutions, with the fastest effects occurring at a 1:7 and 1:8 dilutions. When used at multiple dilutions (1:6, 1:8, 1:9, and 1:10; Figure 5), INCO caused a decrease in fibroblast length almost immediately upon administration (between 0‐1 hours). Unlike ABO, at a 1:7 dilution, INCO caused fibroblast contraction and shortening between 2 to 4 hours after administration. At a 1:8, 1:9, or 1:10 INCO dilutions, contraction and shortening were detected between 0 and 1 hour postadministration. LETI caused fibroblast contraction at all dilutions except 1:9 (Figure 6), with contraction commencing between 0 to 1 hour postadministration of 1:6, 1:7, 1:8, and 1:10 LETI dilutions. At 1:4, LETI caused fibroblast shortening from 1 hour onwards.

**Figure 5:**
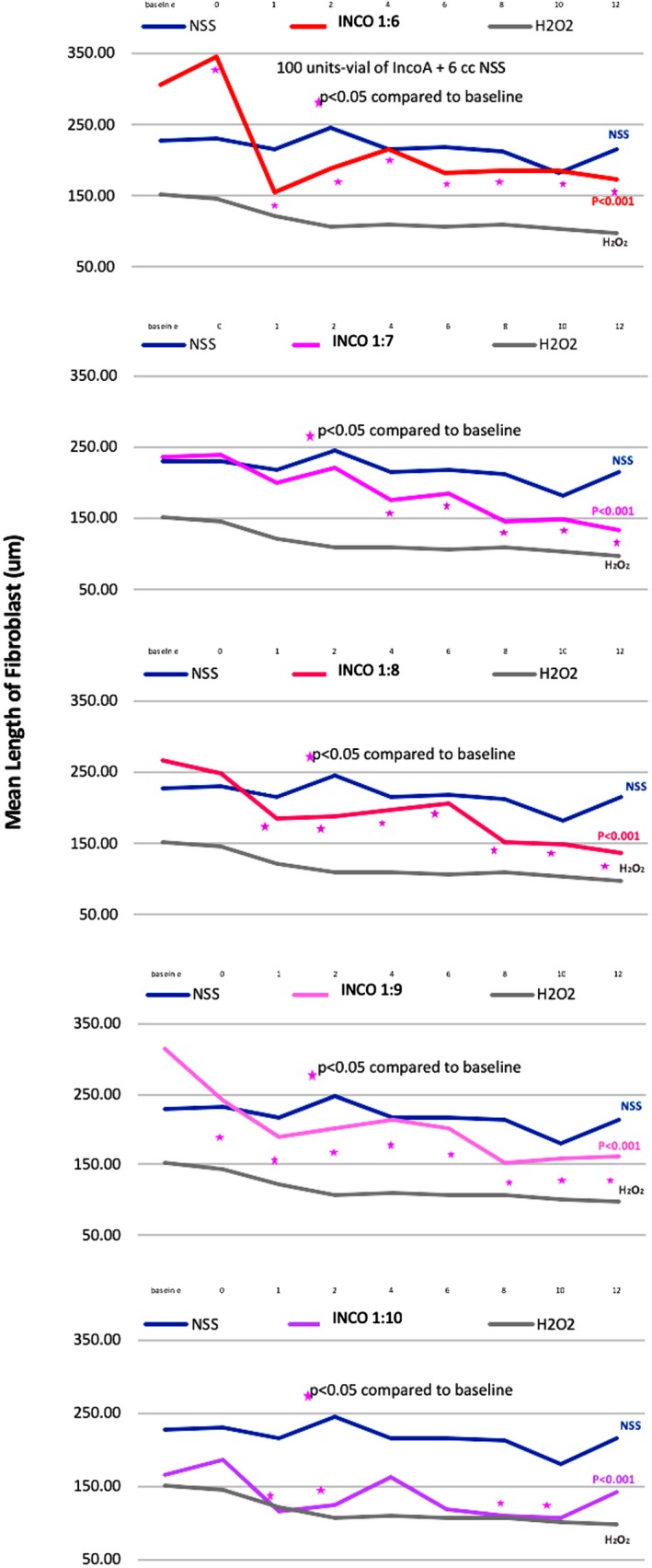
Mean length of INCO‐treated fibroblasts. INCO was diluted 1:6, 1:7, 1:8, 1:9, and 1:10 and analyzed together with fibroblasts treated with NSS or H_2_O_2_ controls

**Figure 6:**
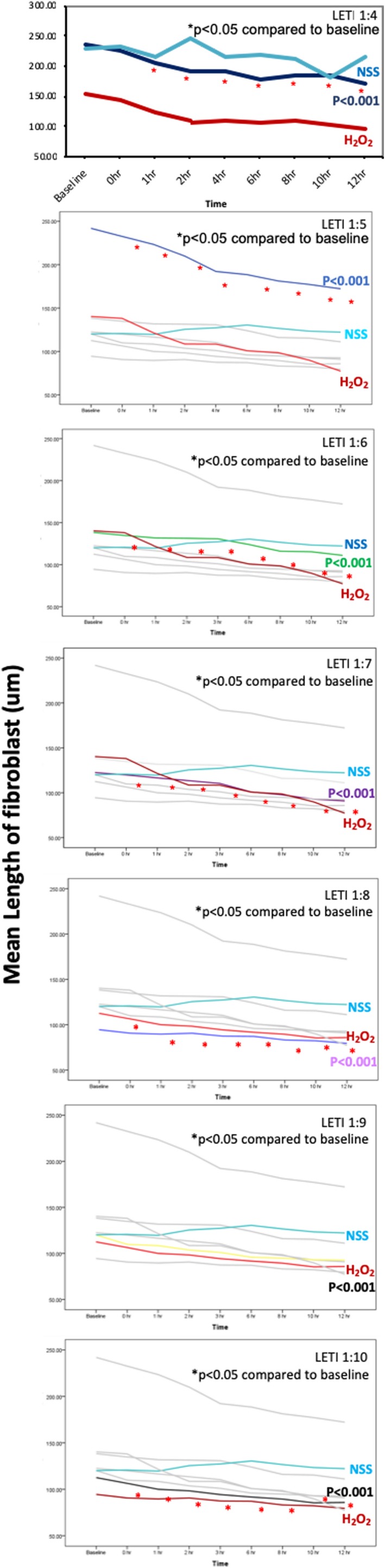
Mean length of LETI‐treated fibroblasts. LETI was diluted 1:4, 1:6, 1:7, 1:8, 1:9, and 1:10 and analyzed together with fibroblasts treated with NSS or H_2_O_2_ controls

## 4 DISCUSSION

To the best of our knowledge, this is the first report that demonstrates the effect of different types and dilutions of BoNT‐A toxin on fibroblast contraction. In previous comparisons of ONA to ABO, statistically significant improvements were observed for forehead lines, glabellar frown lines, and crow's feet compared with placebo groups,^38^, ^39^, ^40^, ^41^, ^42^, ^43^, ^44^, ^45^, ^46^, ^47^, ^48^ and a recent comparison of intradermal versus intramuscular BoNT‐A by Sapra et al showed significantly improved skin texture concomitant with a mild midfacial lift. We propose that one potential cellular mechanism underlying this positive outcome is the fibroblast contraction observed here.

Toxin dilutions have not previously been compared in this manner. In our study, we experimented with dilutions that we routinely use in our day‐to‐day clinical practice. For example, for intradermal injections in our patients, we usually deliver ONA and several other toxin brands, by using a 1:4 to 1:10 dilution, whereas ABO is diluted 1:7.

Although more diluted toxins may seem to produce better and faster fibroblast contraction, in reality, more diluted toxins deliver lower total toxin dosages, thereby reducing toxin efficacy and longevity in clinical practice. We observed that, at a 1:7 dilution ratio, PRABO immediately induced a significant decrease in fibroblast length; however, this speed was not observed at any other PRABO dilution. At 1:7 dilution, ABO also induced significant fibroblast contraction, albeit only after 10‐12 hours while no other dilution demonstrated the same extent of cell shortening. This shortening was not noticeable with other ABO dilutions, at any timepoint. In contrast, ONA did not induce fibroblast contraction, as shown by the lack of any meaningful change in cell length, at any timepoint. Importantly and almost immediately, INCO was the only toxin able to significantly shorten cells at all dilutions investigated. For patients, this shortening translates into potentially near‐immediate lifting effects that may coincide with the entire duration of reported toxin efficacy. INCO's capacity for significant and near‐immediate fibroblast contraction can thus be used a treatment endpoint in clinical settings. In the author's opinion, 1:6 is the most practical dilution for clinical use, considering that pan‐facial toxin injections require a total dosage of 50‐60 U. Using fewer than 50 units for pan‐facial treatments produces no visible lifting effects at 2 weeks, even if some lifting is observed immediately postinjection. In addition, although many dilutions of LETI and PRABO reported here cause fibroblast contractions, most dilutions were greater than 1:6 and expected to be clinically effective only in the short‐term.

We therefore conclude that different BoNT‐A types induce fibroblast contraction to different extents and at different speeds. Whether this was a consequence of the particular toxin type or whether the nontoxin components of the commercial preparations were responsible for this disparity, remains to be established. Of clinical significance was our finding that, while the fibroblasts displayed a measurable decrease in length, their overall size did not change and they did not disappear from the field of view. We therefore believe that BoNT‐A had no cytotoxic effect on fibroblasts, in agreement with the observations by other investigators.^36^ Due to these varying outcomes observed here, physicians should carefully consider the speed at which they hope to achieve an outcome, especially if fibroblast contraction may produce a visible tissue “lifting.”

It would also be interesting to determine and compare the transcriptome and/or proteome profile of human dermal fibroblasts treated with these different botulinum toxins. An in‐depth profile of these changes can provide botulinum toxin users with an understanding of the molecular mechanisms behind the outcomes of different toxin‐based esthetic interventions and also clarify why different commercial preparations produce different results. For example, the mRNAs for collagen 19a1, nitric oxide synthase 2 (NOS2), chromosome 13 open reading frame 15, and FBJ murine osteosarcoma viral oncogene homolog (FOS) were all upregulated in a recent transcriptome analysis of BoNT‐A‐treated human dermal fibroblasts.^49^ In comparison, the expression level of ficolin (collagen/fibrinogen domain containing lectin) 2 (hucolin), E2F transcription factor 1, and baculoviral IAP repeat containing five (BIRC5) was downregulated. The drop in NOS2 levels was thought to be associated with the regulation of cell proliferation, while a rise in FOS levels was linked with the regulation of proliferation and cellular senescence.^50^, ^51^, ^52^, ^53^, ^54^, ^55^ BIRC5 may have participated in reducing apoptosis, while PLAC8 may have regulated the cell cycle and assisted with fibroblast apoptosis and division. Levels of the FGFR3P long noncoding RNA were found to progressively increase in fibroblasts treated with increasing doses of BoNT‐A (from 2.5 U/10^6^ cells to 7.5 U/10^6^ cells),^48^ with a similar concurrent and gradual increase also observed in COL19A1 levels.

Our interpretations would benefit from the analysis of a much larger sampling of cells; currently, our study is limited by the measurement of 20 fibroblast per field of view even though 50 fibroblasts are selected from each dilution. In addition, there are many more dilutions that can be used in practice. Unfortunately, our study was designed to test only particular dilutions of each toxin as listed in Table 1. Our study was entirely based on cell culture, and further investigations need to be performed to establish repeatability—since the cell cultures and individual toxin assays were performed on separate occasions, direct comparisons (e.g. to directly compare PRABO‐ and INCO‐treated fibroblasts) to establish the relative degree of fibroblast shortening were not possible. Although we have performed this same experiment with other toxins at a 1:1 dilution, none yielded a significant contraction except INCO, which produced the greatest contraction within the shortest time (data not shown). As such, further work is needed to directly compare between toxins. Finally, it was also challenging to collect measurements of the same fibroblast over a 24‐hour period due to its proliferation.

## 5 CONCLUSION

To our knowledge, this is the first demonstration of different effects on fibroblast contraction by different commercial preparations of BoNT‐A toxins. We have shown that different types and dilution of BoNT‐A provided variable degree of fibroblast contraction in vitro. Therefore, product selection and dilution used may affect the clinical outcome of intradermal injection of BoNT‐A for face lifting.

## AUTHOR CONTRIBUTIONS

Dr Wanitphakdeedecha had full access to all of the data in the study and takes responsibility for the integrity of data and the accuracy of the data analysis. Study concept and design: Drs. Wanitphakdeedecha and Manuskiatti. Acquisition of data: Drs. Kaewkes, Ungaksornpairote and Ms Limsaengurai. Analysis and interpretation of data: Drs. Wanitphakdeedecha and Kaewkes. Drafting of the manuscript: Dr Wanitphakdeedecha. Critical revision of the manuscript for important intellectual content: Drs. Panich and Manuskiatti. Statistical analysis: Dr Kaewkes. Funding: None. Administrative, technical, or material support: Dr Manuskiatti. Study supervision: Dr Wanitphakdeedecha.

## References

[1] Walker TJ , Dayan SH . Comparison and overview of currently available neurotoxins. J Clin Aesthet Dermatol. 2014;7(2):31‐39. PMC3935649 24587850

[2] Ebrahimi A , Radmanesh M . Botulinum toxin type‐A (BT‐A) for the treatment of multiple eccrine hydrocystomas. J Dermatolog Treat. 2010;21:80‐82. 19363739 10.1080/09546630902877907

[3] Neumeister MW , Chambers CB , Herron MS , et al. Botox therapy for ischemic digits. Plast Reconstr Surg. 2009;124:191‐201. 19568080 10.1097/PRS.0b013e3181a80576

[4] Naik HB , Steinberg SM , Middelton LA , et al. Efficacy of intralesional botulinum toxin A for treatment of painful cutaneous leiomyomas: a randomized clinical trial. JAMA Dermatol. 2015;151:1096‐1102. 26244563 10.1001/jamadermatol.2015.1793 PMC7712636

[5] Bonaparte JP , Ellis D . Alterations in the elasticity, pliability, and viscoelastic properties of facial skin after injection of onabotulinum toxin A. JAMA Facial Plast Surg. 2015;17:256‐263. 25996589 10.1001/jamafacial.2015.0376

[6] Rose AE , Goldberg DJ . Safety and efficacy of intradermal injection of botulinum toxin for the treatment of oily skin. Dermatol Surg. 2013;39:443‐448. 23293895 10.1111/dsu.12097

[7] Shah AR . Use of intradermal botulinum toxin to reduce sebum production and facial pore size. J Drugs Dermatol. 2008;7:847‐850. 19112798

[8] Caire ML , Suskind DL , Tilton AH . Botulinum toxin in the treatment or prevention of acne. Google PatentsPCT/US2002/023670, 2003.

[9] Li ZJ , Park SB , Sohn KC , et al. Regulation of lipid production by acetylcholine signaling in human sebaceous glands. J Dermatol Sci. 2013;72:116‐122. 23849311 10.1016/j.jdermsci.2013.06.009

[10] Grando SA , Zachary CB . The non‐neuronal and non‐muscular effects of botulinum toxin: an opportunity for a deadly molecule to treat disease in the skin and beyond. Br J Dermatol. 2018;178(5):1011‐1019. 29086923 10.1111/bjd.16080

[11] Blomberg M , Jeppesen EM , Skovby F , Benfeldt E . FGFR3 mutations and the skin: report of a patient with a FGFR3 gene mutation, acanthosis nigricans, hypochondroplasia and hyperinsulinemia and review of the literature. Dermatology. 2010;220:297‐305. 20453470 10.1159/000297575

[12] Lee CJ , Lee MH , Cho YY . Fibroblast and epidermal growth factors utilize different signalling pathways to induce anchorage‐independent cell transformation in JB6 Cl41 mouse skin epidermal cells. J Cancer Prev. 2014;19:199‐208. 25337589 10.15430/JCP.2014.19.3.199 PMC4189506

[13] Arnold PB , Fang T , Songcharoen SJ , Ziakas G , Zhang F . Inflammatory response and survival of pedicled abdominal flaps in a rat model after perivascular application of botulinum toxin type A. Plast Reconstr Surg. 2014;133:491e–498e. 10.1097/PRS.0000000000000030 24352212

[14] Park TH , Lee SH , Park YJ , et al. Presurgical botulinum toxin A treatment increases angiogenesis by hypoxia‐inducible factor‐1a/vascular endothelial growth factor and subsequent superiorly based transverse rectus abdominis myocutaneous flap survival in a rat model. Ann Plastic Surg. 2016;76:723‐728. 10.1097/SAP.0000000000000435 25695458

[15] Kim TK , Oh EJ , Chung JY , Park JW , Cho BC , Chung HY . The effects of botulinum toxin A on the survival of a random cutaneous flap. J Plast Reconstr Aesthet Surg. 2009;62:906‐913. 18436495 10.1016/j.bjps.2007.12.034

[16] Bandala C , Teran‐Melo JL , Anaya‐Ruiz M , et al. Effect of botulinum neurotoxin type A (BoNTA) on the morphology and viability of 3T3 murine fibroblasts. Int J Clin Exp Pathol. 2015;8:9458‐9462. 26464704 PMC4583936

[17] Permatasari F , Hu Y‐Y , Zhang J‐A , Zhou B‐R , Luo D . Anti‐photoaging potential of botulinum toxin type A in UVB‐induced premature senescence of human dermal fibroblasts in vitro through decreasing senescence‐related proteins. J Photochem Photobiol B. 2014;5(133):115‐123. 10.1016/j.jphotobiol.2014.03.009 24727404

[18] Kim S , Ahn M , Piao Y , et al. Effect of botulinum toxin type A on TGF‐b/Smad pathway signaling: implications for silicone‐induced capsule formation. Plast Reconstr Surg. 2016;138:821e‐829e. 10.1097/PRS.0000000000002625 27391832

[19] Oh S‐H , Lee Y , Seo Y‐J , et al. The potential effect of botulinum toxin type A on human dermal fibroblasts: an in vitro study. Dermatol Surg. 2012;38:1689‐1694. 22742715 10.1111/j.1524-4725.2012.02504.x

[20] Lee SD , Yi M‐H , Kim DW , Lee Y , Choi Y , Oh S‐H . The effect of botulinum neurotoxin type A on capsule formation around silicone implants: the in vivo and in vitro study. Int Wound J. 2016;13:65–71. 24602064 10.1111/iwj.12228 PMC7949769

[21] Kucukkaya D , Irkoren S , Ozkan S , Sivrioglu N . The effects of botulinum toxin A on the wound and skin graft contraction. J Craniofac Surg. 2014;25:1908‐1911. 25102391 10.1097/SCS.0000000000000941

[22] Abdallah Hajj Hussein I , Dali Balta N , Jurjus RA , et al. Rat model of burn wound healing: effect of botox. J Biol Regul Homeost Agents. 2012;26:389‐400. 23034258

[23] Xiao Z , Qu G . Effects of botulinum toxin type A on collagen deposition in hypertrophic scars. Molecules. 2012;17:2169‐2177. 22354193 10.3390/molecules17022169 PMC6268678

[24] Park TH , Park JH , Chang CH , Rah DK . Botulinum toxin A upregulates Rac1, Cdc42, and RhoA gene expression in a dose‐dependent manner: in vivo and in vitro study. J Craniofac Surg. 2016;27:516‐520. 26963302 10.1097/SCS.0000000000002272

[25] Jeong HS , Lee BH , Sung HM , et al. Effect of botulinum toxin type A on differentiation of fibroblasts derived from scar tissue. Plast Reconstr Surg. 2015;136:171e–178e. 10.1097/PRS.0000000000001438 26218391

[26] Xiaoxue W , Xi C , Zhibo X . Effects of botulinum toxin type A on expression of genes in keloid fibroblasts. Aesthet Surg J. 2014;34:154‐159. 23709452 10.1177/1090820X13482938

[27] Yan T , Chen M , Ma K et al. [Effects of botulinum toxin type A on the expression of alpha‐SMA and myosin‐II of fibroblasts in scars]. Zhonghua Zheng Xing Wai Ke Za Zhi. 2014;30:118‐121. 24941763

[28] Chen M , Yan T , Ma K , et al. Botulinum toxin type A inhibits a smooth muscle actin and myosin II expression in fibroblasts derived from scar contracture. Ann Plast Surg. 2016;77:e46‐e49. 25144422 10.1097/SAP.0000000000000268

[29] Chang S‐P , Tsai H‐H , Chen W‐Y , Lee W‐R , Chen P‐L , Tsai T‐H . The wrinkles soothing effect on the middle and lower face by intradermal injection of botulinum toxin type A. Int J Dermatol. 2008;47:1287‐1294. 19126019 10.1111/j.1365-4632.2008.03895.x

[30] Carruthers A , Carruthers J . Clinical indications and injection technique for the cosmetic use of botulinum A exotoxin. Dermatol Surg. 1998;24(11):1189‐1194. 9834738 10.1111/j.1524-4725.1998.tb04097.x

[31] Petchngaovilai C . Midface lifting with botulinum toxin: intradermal technique. J Cosmet Dermatol. 2009;8(4):312‐316. 19958437 10.1111/j.1473-2165.2009.00467.x

[32] Sapra P , Demay S , Sapra S , et al. A single‐blind, split‐face, randomized, pilot study comparing the effects of intradermal and intramuscular injection of two commercially available botulinum toxin A formulas to reduce signs of facial aging. J Clin Aesthet Dermatol. 2017;10:34‐44. PMC5367871 28367260

[33] Wanitphakdeedecha R , Ungaksornpairote C , Kaewkes A , Rojanavanich V , Phothong W , Manuskiatti W . The comparison between intradermal injection of abobotulinumtoxinA and normal saline for face‐lifting: a split‐face randomized controlled trial. J Cosmet Dermatol. 2016;15:452‐457. 27647769 10.1111/jocd.12289

[34] Kapoor R , Shome D , Jain V , Dikshit R . Facial rejuvenation after intra‐ dermal botulinum toxin: is it really the botulinum toxin or is it the pricks? Dermatologic Surg. 2010;36(Suppl. 4):2098‐2105. 10.1111/j.1524-4725.2010.01703.x 21134041

[35] Sirithanabadeekul P , Lapsomboonsiri S , Rungjang A , Thanasarnaksorn W . Split face comparison between common concentration vs double dilution of intradermal abobotulinum toxin type A (Dysport) injection for facial lifting in Asians. J Cosmet Dermatol. 2018;17:355‐360. 29878718 10.1111/jocd.12667

[36] Cohen S , Artzi O , Heller L . Forehead lift using botulinum toxin. Aesthet Surg J. 2018;38(3):312‐320. 29040367 10.1093/asj/sjx162

[37] Schindelin J , Rueden CT , Hiner MC , Eliceiri KW . The ImageJ ecosystem: an open platform for biomedical image analysis. Mol Reprod Dev. 2015;82(7‐8):518‐529. 26153368 10.1002/mrd.22489 PMC5428984

[38] Nahai F , Lorenc ZP , Kenkel JM , et al. A review of onabotulinumtoxinA (Botox). Aesthet Surg J. 2013;33(1 Suppl):9S‐12S. 23515199 10.1177/1090820X12474629

[39] Nahai F , Lorenc ZP , Kenkel JM , et al. A review of abobotulinumtoxinA (Dysport). Aesthet Surg J. 2013;33(1 Supplement):13S‐17S. 23515194 10.1177/1090820X12474632

[40] Carruthers A , Bruce S , Cox SE , Kane M , Lee E , Gallagher CJ . OnabotulinumtoxinA for treatment of moderate to severe crow’s feet lines: a review. Aesthet Surg J. 2016;36(5):591‐597. 26979457 10.1093/asj/sjw025

[41] Moers‐Carpi M , Carruthers J , Fagien S , et al. Efficacy and safety of onabotulinumtoxinA for treating crow’s feet lines alone or in combination with glabellar lines: a multicentre, randomized, controlled trial. Dermatol Surg. 2015;41(1):102‐112. 25485803 10.1097/DSS.0000000000000220

[42] Carruthers A , Bruce S , de Coninck A , et al. Efficacy and safety of onabotulinumtoxinA for the treatment of crow’s feet lines: a multicentre, randomized, controlled trial. Dermatol Surg. 2014;40(11):1181‐1190. 25347451 10.1097/DSS.0000000000000128

[43] Solish N , Rivers JK , Humphrey S , et al. Efficacy and safety of onabotulinumtoxinA treatment of forehead lines: a multicentre, randomized, dose‐ranging controlled trial. Dermatol Surg. 2016;42(3):410‐419. 26863598 10.1097/DSS.0000000000000626

[44] Dayan S , Coleman WP , Dover JS , et al. Effects of onabotulinumtoxinA treatment for crow’s feet lines on patient‐reported outcomes. Dermatol Surg. 2015;41(Suppl 1):S67‐S74. 25548848 10.1097/DSS.0000000000000146

[45] Kane MA , Brandt F , Rohrich RJ , et al. Evaluation of variable‐dose treatment with a new U.S. botulinum toxin type A (Dysport) for correction of moderate to severe glabellar lines: results from a phase 3, randomized, double‐blind, placebo‐controlled study. Plast Reconstr Surg. 2009;124(5):1619‐1629. 19584772 10.1097/PRS.0b013e3181b5641b

[46] Moy R , Maas C , Monheit G , et al. Long‐term safety and efficacy of a new botulinum toxin type A in treating glabellar lines. Arch Facial Plast Surg. 2009;11(2):77‐83. 19289677 10.1001/archfacial.2009.5

[47] Brandt F , Swanson N , Baumann L , Huber B . Randomized, placebo‐controlled study of a new botulinum toxin type A for treatment of glabellar lines: efficacy and safety. Dermatol Surg. 2009;35(12):1893‐1901. 19549186 10.1111/j.1524-4725.2009.01235.x

[48] Hexsel D , Brum C , Porto MD , et al. Full‐face injections of variable total doses of abobotulinum toxin type A: a randomized, phase IV clinical trial of safety and efficacy. J Drugs Dermatol. 2013;12(12):1356‐1362. 24301236

[49] Miao Y‐Y , Liu J , Zhu J , et al. The Effect of botulinum toxin type A on expression profiling of long noncoding rnas in human dermal fibroblasts. Biomed Res Int. 2017;2017:2957941. 28265570 10.1155/2017/2957941 PMC5318640

[50] Okayama H , Saito M , Oue N , et al. NOS2 enhances KRAS‐induced lung carcinogenesis, inflammation and microRNA‐21 expression. Int J Cancer. 2013;132(1):9‐18. 22618808 10.1002/ijc.27644 PMC3473150

[51] Haag D , Zipper P , Westrich V , et al. Nos2 inactivation promotes the development of medulloblastoma in Ptch+/− mice by deregulation of Gap43‐dependent granule cell precursor migration. PLoS Genet. 2012;8(3):e1002572. 22438824 10.1371/journal.pgen.1002572 PMC3305407

[52] Lee CS , Bae I‐H , Han J , et al. Compound K inhibits MMP‐1 expression through suppression of c‐Src‐dependent ERK activation in TNF‐α‐stimulated dermal fibroblast. Exp Dermatol. 2014;23(11):819‐824. 25181017 10.1111/exd.12536

[53] Lephart ED . Protective effects of equol and their polyphenolic isomers against dermal aging: microarray/protein evidence with clinical implications and unique delivery into human skin. Pharmaceutical Biology. 2013;51(11):1393‐1400. 23862588 10.3109/13880209.2013.793720

[54] Hwang YP , Choi JH , Kim HG , et al. Cultivated ginseng suppresses ultraviolet B‐induced collagenase activation via mitogen‐activated protein kinases and nuclear factor κB/activator protein‐1‐dependent signaling in human dermal fibroblasts. Nutrition Research. 2012;32(6):428‐438. 22749179 10.1016/j.nutres.2012.04.005

[55] Quan T , Qin Z , Xu Y , et al. Ultraviolet irradiation induces CYR61/CCN1, a mediator of collagen homeostasis, through activation of transcription factor AP‐1 in human skin fibroblasts. J Invest Dermatol. 2010;130(6):1697‐1706. 20164845 10.1038/jid.2010.29

